# The Power of Ethnotheories in Changing Societies: Commentary on Cross‐Cultural Research on Parents: Applications to the Care and Education of Children

**DOI:** 10.1002/cad.20342

**Published:** 2020-05-28

**Authors:** Paul P. M. Leseman

**Affiliations:** ^1^ Utrecht University

The research reported in the papers of this issue reveals the power of parental ethnotheories in regulating basic biological processes, genetic predispositions and, in general, in orchestrating child development to optimally fit the local circumstances, including institutional settings such as preschools and schools. Crucial to this power is the alignment with broader cultural themes in the wider society, the frequent repetition of the key messages by different persons, the incorporation of these messages in continuous discourse among parents, and the alignment of parental and professional discourses. Ethnotheories, as they are supported and embedded in communities, are strong regulators of development, and whatever be their precise content, strong ethnotheories support optimal development. The strength of proximal processes in child development to actualize potential depends on the consistency of these processes over time and on the coherence of these processes across the microsystems of the child (Bronfenbrenner & Morris, 2006).

The “three R's” of Dutch child rearing is an example. It is not in an absolute sense that this ethnotheory is conducive to optimal child development—not more than other ethnotheories—but it is the strength of the model and the attunement of other ecological contexts, in addition to the family, to it (e.g. the acceptance of part‐time work for mothers) that makes the “three R's” a powerful force. One could wonder whether the earlier regular diurnal biorhythms in Dutch infants as compared to U.S. infants results in better physical and mental health (less childhood disease, lower prevalence of ADHD), but evidence is lacking. Whatever the facts of the matter, the paper by Van Schaik and colleagues (Van Schaik et al., this issue) suggests that a main gain of the Dutch childrearing (including the strategy of “stretching feeding times”) is that it fits Dutch mothers’ need for some time for themselves, that it allows regular family life with fixed times for daily meals, and that it aligns well with the highly scheduled wider Dutch society (which is still far from a full 24 hours economy as exists in the United States). In the United States, parents with infants and young children also consider regularity—they even hope for it. They know about the concept and see the potential advantages for themselves and for family life, but more than in the Netherlands, they encounter competing models and theories, such as the importance of stimulating children and providing family activities. Also, geographic (longer distances to travel) and economic factors in the studied samples in the United States are less supportive to a highly scheduled regular family life.

The studies on concepts of the difficult child and of the successful school child are particularly revealing (Super et al., this issue; Feng, Harkness, Super, Welles, Rios Bermúdez, Bonichini, Moscardino, & Zylicz, this issue). The findings attest to the urgency of cross‐cultural research in psychology, and provide a strong argument for psychology as a *social* science. While basic components of children's psychological and behavioral profiles are shared among parents in different cultures, suggesting some degree of universalism, the associations of these components with others reveal interesting differences across cultures. The “difficult child” is at least partly a cultural construction. The same basic characteristics, and even similar configurations of characteristics making up a particular child profile, are valued differently depending on the interplay of children's age and the cultural and institutional context. This should remind us that universals in child development may exist at a basic psychobiological level, but disappear when it comes to more complex configurations of biological, psychological, and behavioral characteristics as children develop. Further, it has implications for designing curricula for early childhood programs. Implementing programs developed and proven effective in one culture may fail in another culture because of a mismatch with the ethnotheories on desirable competences of this other culture, as is reported for Botswana (Tsamaase, Harkness, & Super, this issue).

The paper by Feng et al. (this issue) also should hold up a mirror to psychologists who try to capture development on one‐dimensional scales of “normal development,” from which some children deviate. Trying to classify “difficult, less than ideal school children” in common classification systems that presumably reflect “real deficits” and non‐social “neurobiological causes,” may be pointless. The trend in many countries to medicalize special education ignores the social construction of the “difficult, less than ideal school child” and disregards the way this construction is partly determined by characteristics of the education system. This approach has not resulted in an overall reduction of the number of children with special needs nor in successful inclusion of these children in mainstream education (Rix, Sheehy, Fletcher‐Campbell, Crisp, & Harper, 2013). Instead, a rise of diagnosed behavior problems and specific learning disabilities (e.g., dyslexia) of growing numbers of “less than ideal school children” has been reported. The medicalization of the child probably also influences parents’ ethnotheories, with the consequence that parents may wish their child to be diagnosed with a behavioral or learning disorder. It is often the only way to be eligible for education that adapts to the child. A cultural‐constructivist model holds better promise for an inclusive education agenda. Finland is an interesting example of a country with such a model, together with a strong equity agenda. It is the country with one of the largest percentages of children with special or additional needs, yet also the country with one of the smallest percentages of children in segregated special schools. Here the cultural model is that all children differ on multiple dimensions, that difficulties result from a mismatch between the child and the mainstream education approach, and that each child has the right to be educated in the way best fitting his or her profile. Finnish education is, therefore, highly inclusive and provides adapted instruction to large numbers of children (Takala, Pirttimaa, & Törmänen, 2009).

The research reported in this issue also points to the dynamic nature of parental ethnotheories, revealing changes as adaptive responses to changing socioeconomic, demographic, institutional, and political circumstances (cf. Valsiner & Litvinovic, 1996). These changes are not always easy and reveal struggles within parents, communities, and societies. Optimal fit is not always achieved nor automatically guaranteed. Changes and struggles reveal the desire to preserve traditions, to stay connected to older generations or to the country of origin, and to reduce uncertainty, while at the same time there is a need to adapt. Parents’ intimate social networks play a critical role in processes of change, but relationships with professionals, as substructures within parents’ social networks, are also important and can serve to connect groups of parents who risk exclusion from society and its institutions at large. Networks are learning communities, as de Haan, Koeman, and de Winter (this issue) illustrate. For migrant mothers, their social networks play an important role in adapting the traditional ethnotheories of child rearing to the post‐migration situation. The de Haan et al. paper also shows that networks differ in structure and composition, and may not be equally supportive of adaptive changes in parental ethnotheories. Professionals such as preschool teachers and youth health care workers have a key role to play here. Trusting relationships between parents and professionals are pivotal to integration. Culturally sensitive professionals and inclusive practices of care and education can prevent dissociations in parents’ networks between the in‐group subclusters and subclusters with out‐group (mainstream) professionals.

Ethnotheories follow changes in society without fully breaking with tradition, resulting in new, potentially productive views and socialization strategies, while maintaining old wisdom. The study of the cultural model of shyness in current China is exemplary (Liu, Harkness, & Super, this issue). Whereas shyness in Western models is often regarded as problematic and equated with withdrawn, internalizing and anxious avoidant behavior, modern Chinese middle‐class parents have developed a more differentiated concept with a positive value, also acknowledged by teachers, in which traditional Confucian values of modesty, reserve, attentiveness, and good manners are seamlessly integrated with western values of assertiveness, independence, and communicative competence. The new values reflect what parents deem essential skills in the market‐oriented, competitive new Chinese economy. Although apparently contradictory, smooth integration of these different values is possible according to the interviewees, based on the belief that shyness and assertiveness are malleable. A shy child can be supported to become more assertive, autonomous, and communicative, while maintaining the positively valued characteristics of shyness. This theory guides new child rearing practices. Compared to their own childhood, the Chinese mothers in the Liu et al. study allow children more initiative and disobedience, and they put more emphasis on communication and cognitive stimulation. Yet, interestingly, this renewed concept of shyness also seems to counterbalance a too strong emphasis on academic competitiveness, as several of the Chinese parents expressed their concern about the increasing academic pressure. The focus groups with grandparents in Botswana, partly coming from a small traditional town and partly from a large modern city, show similar adaptation processes in parental ethnotheories related to the rural‐urban divide (Tsamaase et al., this issue). Urban grandmothers expect independent and responsible behavior at a later age than rural grandmothers, and their socialization practices differ, for instance with regard to talking with children instead of instructing them. Yet, their understanding of “clever” remains associated with traditional notions of social responsibility and social competence.

An interesting finding is the regional‐boundedness of ethnotheories and, regarding the views of parents on preschool practices, the divide between the European social‐pedagogy tradition and the U.S. approach (Feng et al., this issue; Harkness et al., this issue). In Europe, countries in the South are more similar to each other than to other countries, and the same holds for countries clustering in the North or the East. Anglo‐Saxon countries also form a cluster that differs from all European clusters studied. It is interesting to speculate about the background of this pattern of similarities and differences, considering the role of family structure, religious institutions, and the nature of the welfare state. The extended‐family structure in the South and the dominance of the collectivistic Roman Catholic Church are long‐standing institutional influences that differ markedly from the core family type and the dominance of the Protestant churches in the North. The predominant social‐democratic and conservative welfare states of Europe, with still relatively large public sectors in education, health, and social welfare, contrast with the liberal governance tradition and more strongly marketized public sectors of the Anglo‐Saxon countries, including the United States (Esping‐Andersen, 2015). This contrast underscores that parental ethnotheories are interwoven with encompassing networks of cultural meanings and national institutions. In the study of parent–preschool relationships, parents in all studied European countries emphasize social‐emotional development and peer‐relations as primary goals of the preschool program (Harkness et al., this issue). They advocate play‐based learning and good relationships with teachers. They talk a lot with the teachers and other parents, and the preschool is almost an extension of the intimate family home environment: a safe, continuous community space where everyone is included, and where teachers and parents agree upon curriculum and pedagogy. The U.S. sample shows a different pattern. Preschools present, more than in the European countries, a human capital agenda, promoting academic development and teacher‐directed learning. The relationships between parents and teachers are less symmetrical, parents and teachers talk less to each other, and parents are keen, seeing themselves as co‐teachers, to prepare children at home for educational activities in the preschool and onward through primary school.

Doing cross‐cultural research is necessary but difficult. The meaning of concepts, the connotations they evoke, the situatedness of these meanings in the local social and institutional context, make comparisons often difficult and even meaningless unless the research is carefully conducted. The combination of qualitative and ethnographic research with quantitative research using well‐designed questionnaires as reported in this issue, is a convincing solution. Reading the reports of the in‐depth interviews and focus groups, parents’ narratives and perspectives make sense to a reader from a different culture, although one might not necessarily agree with the solutions that parents propose. The papers in this issue suggest that the semantics of the words used is not the primary problem. Apparently, people across cultures share a language to talk about issues of child development and child rearing. They can discuss different values and goals involved in child rearing, and they recognize similar basic child temperament characteristics. They may weigh them differently, though, as is reflected in the different mean ratings, and they may construct different configurations fitting the local parental discourse and institutionalized practices. Such a notion of a shared language is born out in recent research in Europe. In this research, involving countries of all regions in Europe, rigorous tests of measurement invariance were applied on large sample survey and interview data regarding parental beliefs about preschool education, revealing scalar invariance of most constructs, yet with different means and patterns of importance ratings by country (Broekhuizen, Leseman, Moser, & van Trijp, 2015).

The issue of representativeness of the samples is mentioned several times in this collection of papers. To what extent the observed cultural models and ethnotheories are representative of the wider population in the studied countries is an open question. Several indications suggest they are, at least to a reasonable degree. Patterns over time in cross‐sectional research are also fairly stable within countries, as are the differences among countries. However, countries are rapidly changing and becoming culturally more diverse as new groups settle, global relations increase, and economies continue to change, as is already evidenced in several papers in this issue, with consequences for parental ethnotheories. The role of the internet, social media, and the algorithms that create “bubbles” by selectively distributing information, is definitely a new challenge for research on ethnotheories. Future research on parental ethnotheories could examine variations across communities within broader national and cross‐national contexts, and identify where connections are possible and agreement can be reached. This knowledge can support professionals in education, health, and other social services in weaving social cohesion.
